# *Trans*-Chalcone Attenuates Pain and Inflammation in Experimental Acute Gout Arthritis in Mice

**DOI:** 10.3389/fphar.2018.01123

**Published:** 2018-10-02

**Authors:** Larissa Staurengo-Ferrari, Kenji W. Ruiz-Miyazawa, Felipe A. Pinho-Ribeiro, Victor Fattori, Tiago H. Zaninelli, Stephanie Badaro-Garcia, Sergio M. Borghi, Thacyana T. Carvalho, Jose C. Alves-Filho, Thiago M. Cunha, Fernando Q. Cunha, Rubia Casagrande, Waldiceu A. Verri

**Affiliations:** ^1^Departamento de Ciências Patológicas, Universidade Estadual de Londrina, Londrina, Brazil; ^2^Department of Pharmacology, Ribeirão Preto Medical School, University of São Paulo, Ribeirão Preto, Brazil; ^3^Departamento de Ciências Farmacêuticas, Universidade Estadual de Londrina, Londrina, Brazil

**Keywords:** *trans*-Chalcone, joint pain, gouty arthritis, gout flare, inflammation, flavonoids, natural products, rheumatic disease

## Abstract

Gouty arthritis is characterized by an intense inflammatory response to monosodium urate crystals (MSU), which induces severe pain and reduction in the life quality of patients. *Trans*-Chalcone (1,3-diphenyl-2-propen-1-one) is a flavonoid precursor presenting biological activities such as anti-inflammatory and antioxidant proprieties. Thus, the aim of this work was to evaluate the protective effects of *trans*-Chalcone in experimental gout arthritis in mice. Mice were treated with *trans*-Chalcone (3, 10, or 30 mg/kg, per oral) or vehicle (Tween 80 20% plus saline) 30 min before intra-articular injection of MSU (100 μg/knee joint, intra-articular). We observed that *trans*-Chalcone inhibited MSU-induced mechanical hyperalgesia, edema, and leukocyte recruitment (total leukocytes, neutrophils, and mononuclear cells) in a dose-dependent manner. *Trans*-Chalcone also decreased inflammatory cell recruitment as observed in Hematoxylin and Eosin (HE) staining and the intensity of fluorescence of LysM-eGFP+ cells in the confocal microscopy. *Trans*-Chalcone reduced MSU-induced oxidative stress as observed by an increase in the antioxidant defense [Glutathione (GSH), Ferric Reducing (FRAP), and 2,2’-Azinobis-3-ethylbenzothiazoline 6-sulfonic acid (ABTS assays)] and reduction in reactive oxygen and nitrogen species production [superoxide anion (NBT assay) and nitrite (NO assay)]. Furthermore, it reduced *in vivo* MSU-induced interleukin-1β (IL-1β), Tumor necrosis factor-α (TNF-α), and IL-6 production, and increased Transforming growth factor-β (TGF-β) production. Importantly, *trans*-Chalcone reduced nuclear factor kappa-light-chain-enhancer of activated B cells (NF-κB) activation and thereby the mRNA expression of the inflammasome components *Nlrp3* (cryopyrin), *Asc* (apoptosis-associated speck-like protein containing a CARD), *Pro-caspase-1* and *Pro-IL-1β*. *In vitro*, *trans*-Chalcone reduced the MSU-induced release of IL-1β in lipopolysaccharide (LPS)-primed macrophages. Therefore, the pharmacological effects of *trans*-Chalcone indicate its therapeutic potential as an analgesic and anti-inflammatory flavonoid for the treatment of gout.

## Introduction

Gout is a painful inflammatory disease caused by the over-production of uric acid, which forms deposits and crystallizes as monosodium urate (MSU) in articular and periarticular tissues (Rees et al., 2014; Fattori et al., 2016). MSU crystals trigger an intense inflammatory response and increase the recruitment of neutrophils to the joint (Rees et al., 2014; Fattori et al., 2016). Management of gout arthritis lies on the use of urate-lowering therapies and the control of acute flares. The main reason for patient seeking of medical care is the intense pain in acute flares (Rees et al., 2014). Gout flares in humans are self-limited and last for nearly 10 days with intense and debilitating pain despite anti-inflammatory treatment (Martin and Harper, 2010). Nevertheless, if left untreated, continuing MSU crystal deposition causes repetitive flares (Rees et al., 2014; Dalbeth et al., 2016). More importantly, irreversible joint damage with chronic symptoms and disability can be observed. In fact, advanced cases of gout are characterized by chronic joint pain, movement limitation, and recurrent flares (Dalbeth et al., 2016). Currently, the management of gout flares lies on the use of non-steroidal anti-inflammatory drugs (NSAIDs), colchicine, corticoids, or biological agents (Rees et al., 2014; Dalbeth et al., 2016). However, the use of these drugs lack safety in patients with comorbidities (NSAIDs), often cause severe side effects (NSAIDs, colchicine, and corticoids), present high cost (biological agents), or possess non-satisfactory analgesic effects in some patients (Rees et al., 2014; Dalbeth et al., 2016).

Monosodium urate crystals trigger an inflammatory response that is dependent on Nacht, LRR, and Pyd domains-containing protein 3 (NLRP3) inflammasome assembly and production of mature interleukin-1β (IL-1β) (Martinon et al., 2006; Amaral et al., 2012). In addition to its hyperalgesic effect, IL-1β also induces the recruitment of neutrophils that further produce hyperalgesic mediators, such as endothelin-1 and prostaglandin E_2_ (PGE_2_) (Popa-Nita et al., 2007; Cunha et al., 2008; Amaral et al., 2012; Fattori et al., 2016). In neutrophils, IL-1β induces PGE_2_ production, which is a molecule responsible for sensitization of nociceptor sensory neurons (Cunha et al., 2008). Thus, the advances in the understanding of the pathophysiology of pain and inflammation in gout raised the possibility of more effective analgesic therapies.

Flavonoids are natural compounds and represent a major subset of polyphenols widely distributed in vegetables, fruits, and herbs (Verri et al., 2012). In general, there is evidence that flavonoids present broad biological activity such as antioxidant, antitumoral, antimicrobial, anti-inflammatory, and analgesic (Verri et al., 2012). *Trans*-Chalcone (1,3-diphenyl-2-propen-1-one) is an open-chain flavonoid and a major precursor of other flavonoids in plants, which also possesses anti-inflammatory and antioxidant properties (Lamoke et al., 2011; Singh et al., 2016; Martinez et al., 2017a,b). Chalcones have a good pharmacological profile and low toxicity (Karimi-Sales et al., 2018) supporting these molecules as good therapeutic candidates for chronic inflammation such as the observed in rheumatic diseases.

Evidence shows that *trans*-Chalcone reduces UV-induced skin inflammation (Martinez et al., 2016, 2017b), ameliorates carbon tetrachloride (CCl_4_)-induced liver damage (Singh et al., 2016), and prevents pathological neovascularization in the ischemic retina (Lamoke et al., 2011). However, to the date, there is no study addressing the analgesic effect of *trans*-Chalcone or even its effectiveness in gout arthritis. Given the evidence on the biological activities of *trans*-Chalcone and lack of data evaluating its analgesic effect, we aimed at evaluating the efficacy of this flavonoid in a model of MSU-induced gout arthritis in mice.

## Materials and Methods

### Animals and Ethics Statement

Male Swiss (25–30 g, 8 weeks) and C57BL/6 (20–25 g, 8 weeks) mice from the Universidade Estadual de Londrina (Londrina, Brazil), LysM-GFP mice (20–25 g, 8 weeks) and C57/BL6 from the Ribeirão Preto Medical School – University of São Paulo (Ribeirão Preto, São Paulo, Brazil) were used in this study. Mice were housed in standard clear plastic cages with free access to food and water with a light/dark cycle of 12/12 h temperature of 21 ± 1°C. All behavioral testing was performed between 9 a.m. and 5 p.m. in a temperature-controlled (21 ± 1°C) room. Animal care and handling procedures were approved by the Ethics Committee of the Universidade Estadual de Londrina (process number 14600.2013.73). All efforts were made to minimize animal suffering and to reduce the number of animals used.

### Experimental Procedures

Mice were treated with *trans*-Chalcone (TC; 3, 10, or 30 mg/kg, p.o.) or vehicle (Tween 80 20% plus saline) 30 min before intra-articular stimulus with MSU (100 μg/10μL, i.a.). *trans*-Chalcone (at 95% purity was purchased from Santa Cruz Biotechnology, #CAS 614-47-1, Dallas, TX, United States) dose range was selected upon the doses previously used in the literature (Lamoke et al., 2011; Jalalvand et al., 2015; Singh et al., 2016; Karimi-Sales et al., 2018). The negative control of stimulus received only intraarticular injection of 10 μl of saline. In experiments that the effect of *trans*-Chalcone was higher than the control responses, the effect of *trans*-Chalcone *per se* was also investigated.

Mechanical hyperalgesia and edema were evaluated 1, 3, 5, 7, and 15 h after MSU or saline injection. Fifteen hours after MSU injection, knee joint wash was collected to determine leukocyte recruitment. Based on these previous experiments, the dose of 30 mg/kg was chosen for the following experiments: histopathological analysis (HE staining), neutrophil migration to the knee joint wash using LysM-eGFP mice (confocal microscopy), oxidative stress, determination of the expression of genes sensitives to oxidative stress by RT-qPCR (*gp91^phox^*, *Nrf2*, *Ho-1*), cytokine measurement by ELISA, NF-κB activation (total p65/phosphorylated p65 ratio) by ELISA, and inflammasome components expression *Nlrp3*, *Asc*, *Pro-caspase-1*, and *Pro-Il-1β* mRNA expression by RT-qPCR. In all *in vivo* analysis, samples were collected 15 h after MSU injection. Serum samples were also collected 15.5 h after *trans*-Chalcone or vehicle treatment to evaluate renal and hepatic toxicity considering the same time of protocol of the all experiments with *trans*-Chalcone. The negative control received only intraarticular injection of 10 μl of saline. As a positive control for liver injury (hepatic toxicity) and for kidney injury (renal toxicity), acetaminophen (650 mg/kg,) and diclofenac (200 mg/kg) were used, respectively. One protocol evaluated the effect of *trans*-Chalcone (30 mg/kg, p.o) or vehicle (Tween 80 20% plus saline) treatment 30 min after MSU injection on mechanical hyperalgesia and knee edema.

For *in vitro* analysis, to assess the effect of *trans*-Chalcone on NF-κB activation, bone marrow derived macrophages (BMDMs) were pre-treated with 0.1–3 μM before 500 ng/mL of LPS (i.e., priming) and supernatants were collected 5 h after MSU (activation) stimulation and IL-1β levels were quantitated by ELISA. A selected concentration of *trans*-Chalcone using the same protocol was also used to test the flavonoid effect over *Nrf2* and *Ho-1* mRNA expression. Next, it was also evaluated the effect of *trans*-Chalcone at 0.1–3 μM on BMDMs 30 min before MSU stimulation (i.e., after priming with LPS/before activation with MSU) to address if *T*C could interfere directly with the activation stimulus to prevent IL-1β secretion and inflammasome activation. The vehicle (Tween 80 20% plus saline) treatment did not alter the responses. All experiments were performed in Swiss mice except for the experiments presenting LysM-eGFP analysis and BMDM cell culture.

### Preparation of MSU Crystal

The MSU crystals were prepared as described previously (Ruiz-Miyazawa et al., 2017). Briefly, 800 mg of monosodium urate were dissolved in 155 ml of boiling milli-Q water containing 5 ml of NaOH, and after the pH was adjusted to 7.2, the solution was cooled gradually by stirring at room temperature. The crystals were collected, and then centrifuged at 3,000 *g* for 2 min at 4°C. The crystals were evaporated and sterilized by heating at 180°C for 2 h and stored in a sterile environment until use. The load of endotoxin present in the MSU crystals was determined using a ToxinSensor^TM^ Single Test Kit (GenScript). The selected dose and concentration of MSU did not present detectable endotoxin levels as previously described (Ruiz-Miyazawa et al., 2018).

### Induction of MSU-Induced Knee Joint Inflammation

Joint inflammation was induced by the intra-articular (i.a.) administration of MSU (100 μg/10 μl) into the right knee joint of mice under isoflurane anesthesia. Control animals received an i.a. injection of sterile saline (10 μL) (Ruiz-Miyazawa et al., 2017).

### Evaluation of Knee Joint Hyperalgesia

The mechanical hyperalgesia of the femur-tibial joint was evaluated by an electronic von Frey apparatus. Mice were placed in acrylic cages with a wire grid floor, and the stimulations were performed only when the animals were quiet (and with the four paws on the grid floor). This method consists of an electronic pressure-meter, with a force transducer fitted with polypropylene tip (Insight instruments, Ribeirao Preto, Brazil). To evaluate the articular pain, we used a large tip (4.15 mm^2^), to exclude the subcutaneous effect (Guerrero et al., 2006). An increase perpendicular force was applied to the central area of the plantar surface of the hind paw to induce flexion of the femur-tibial joint followed by the hind paw withdrawal. A digital analgesimeter recorded the intensity of the force applied (in grams) when the paw was withdrawal. The test was performed at the times indicated on figures. The investigators were blinded to the treatment groups.

### Knee Joint Edema Evaluation

Knee joint edema was determined with a caliper (Mitutoyo) before (zero time), and after the intra-articular injection with MSU at the times indicated. Knee joint edema was determined for each mouse by the difference between the time points indicated on figures and the zero time (Ruiz-Miyazawa et al., 2017). The edema value is represented as Δmm/joint.

### *In vivo* Leukocyte Migration

Knee joint wash was collected 15 h after MSU injection for determination of leukocyte recruitment (Verri et al., 2010). Briefly, articular cavities were washed three times with 3.3 μL of saline with 1 mM EDTA, then, diluted to a final volume of 50 μL with PBS/EDTA to evaluate leukocyte migration. The total number of leukocytes was determined in a Neubauer chamber diluted in Turk’s solution 1:2 (used to lyse the erythrocytes). Differential cell counts were determined in Rosenfeld stained slices using a light microscope and results are expressed as the number of neutrophils per cavity.

### Histopathological Analysis

Knee joint was collected 15 h after MSU injection, fixed with 10% paraformaldehyde in PBS, and then decalcified for 10 days with EDTA and embedded in paraffin for histological analysis. The paraffin sections were stained with hematoxylin and eosin for conventional morphological evaluation. Results are expressed as leukocytes infiltrate (cell/field) counted at the inflammatory foci as indicative of synovitis. A dimension used for the analysis of the slices was 569 × 633 pixels and magnification ×400 (Ruiz-Miyazawa et al., 2017).

### Fluorescence Assay

Knee joint wash of LysM-GFP mice was collected in sterile slides 15 h after MSU injection into the knee joints. DAPI fluorescent stain was added to slides for localization of nucleus in each sample. The representative images and quantitative analysis were performed using a confocal microscope (SP8, Leica Microsystems, Mannheim, Germany). The intensity of fluorescence was quantified in randomly selected fields of different groups by an investigator blinded to the treatments. Results are presented as the percentage of GFP fluorescent intensity.

### GSH Levels Measurement

Samples of articular joint were collected and maintained at -80°C for at least 48 h. The sample was homogenized with 200 μl of 0.02 M EDTA. The homogenate was mixed with 25 μl of trichloroacetic acid 50% and was homogenized three times over 15 min. The mixture was centrifuged (15 min × 1500 *g* × 4°C). The supernatant was added to 200 μl of 0.2 M TRIS buffer, pH 8.2, and 10 μl of 0.01 M DTNB. After 5 min, the absorbance was measured at 412 nm (Multiskan GO, Thermo Fisher Scientific) against a blank reagent with no supernatant. A standard GSH curve was formed. The results are expressed as GSH per mg of protein (Ruiz-Miyazawa et al., 2017).

### ABTS and FRAP Assays

The ability of samples to resist oxidative damage was determined by their free radical scavenging (ABTS [2,2’-Azinobis-3-ethylbenzothiazoline 6-sulfonic acid] assay) and ferric reducing (FRAP assay) properties. The ABTS is effective in detecting anti-radical activity and FRAP detects reducing potential in tissue samples (Menghini et al., 2018). The tests were adapted to a 96-well microplate format as previously described (Ruiz-Miyazawa et al., 2017). Articular tissue samples were collected 15 h after MSU i.a. injection (100 μg/10 μL) and homogenized immediately in ice-cold KCl buffer (500 μl, 1.15% w/v). The homogenates were centrifuged (200 *g* × 10 min × 4°C), and the supernatants were used in both assays. Diluted ABTS solution (200 μl) was mixed with 10 μL of sample in each well. After 6 min of incubation at 25°C, the absorbance was measured at 730 nm. For FRAP assay, the supernatants (10 μl) were mixed with the freshly prepared FRAP reagent (150 μl). The reaction mixture was incubated at 37°C for 30 min, and the absorbance was measured at 595 nm (Multiskan GO Thermo Fisher Scientific). The results of ABTS and FRAP assays were equated against a standard Trolox curve (0.02–20 nmol).

### Superoxide Anion Production

The measurement of superoxide anion production in tissue homogenates (10 mg/ml in 1.15% KCl) was performed using the nitroblue tetrazolium (NBT) assay adapted to a microplate as described previously (Ruiz-Miyazawa et al., 2017). The NBT reduction was measured at 600 nm (Multiskan GO, Thermo Fisher Scientific). The tissue weight was used for data normalization.

### Nitrite Production

Samples from knee joint were collected 15 h after MSU injection, homogenized in 500 μl of saline, and nitrite (NO_2_^-^) concentration was determined by the Griess reaction as an indicator of nitric oxide (NO) production (Lima-Junior et al., 2013). Briefly, 100 μl of the homogenate was incubated with 100 μl of Griess reagent for 5 min at 25°C, and NO_2_^-^ concentration was determined by measuring the optical density at 550 nM (Multiskan GO, Thermo Fisher Scientific) in reference to a standard curve of NaNO_2_ solution. Results are expressed as μmol of NO_2_^-^ per mg of tissue.

### Preparation of Bone Marrow-Derived Macrophages (BMDMs)

Bone marrow cells from femora and tibiae of C57BL/6 mice (8 weeks old) were aspirated and flushed with a syringe filled with RPMI 1640 to extrude the bone marrow into a 15 ml sterile polypropylene tube. A 5 ml plastic pipette was used to homogenize the bone marrow. The fresh bone marrow cells obtained were cultured with RPMI 1640 media supplemented with 10% FBS, 2 mM L-glutamine, 100 U/ml penicillin, 100 μg/ml streptomycin and 15% of L929 cell conditioned medium, as source of granulocyte/macrophage colony stimulating factor and seeded in non-tissue culture treated Optilux Petri dishes (BD Biosciences) and incubated at 37°C in a 5% CO_2_ atmosphere for 7 days (Marim et al., 2010).

To obtain the BMDM, the supernatants were discarded and the attached cells were washed with 10 ml of sterile PBS 1×. Ten milliliters of ice-cold PBS were added to each plate and incubated at 4°C for 10 min to detach the cells. The macrophages were then aspirated, counted, seed and cultivated in tissue culture plates for 12 h before further RT-qPCR and ELISA assay.

### Reverse Transcription and Quantitative Polymerase Chain Reaction (RT-qPCR)

Total RNA was extract from 3 × 10^6^ bone marrow macrophages cells (BMDMs) 5 h after MSU activating stimulus and from knee joints at 15 h after MSU injection by using TRIzol^®^ reagent. The total RNA was isolated according to manufacturer’s directions. The purity of total RNA was measured with a spectrophotometer and the wavelength absorption ratio (260/280 nm) was between 1.8 and 2.0 for all preparations. Reverse transcription of total RNA to cDNA and qPCR were carried out using GoTaq^®^ 2-Step RT-qPCR System (Promega) and specific primers (Applied Biosystems^®^). The mRNA level of glyceraldehyde 3-phosphate dehydrogenase (*Gapdh*) was used as reference gene. The primers used were *Gapdh* forward: CAT ACC AGG AAA TGA GCT TG, reverse: ATG ACA TCA AGA AGG TGG TG; *gp91^phox^*, forward: AGC TAT GAG GTG GTG ATG TTA GTG G, reverse: CAC AAT ATT TGT ACC AGA CAG ACT TGA G; *Nrf2*, forward: TCA CAC GAG ATG AGC TTA GGG CAA, reverse: TAC AGT TCT GGG CGG CGA CTT TAT; *Ho-1*, forward: CCC AAA ACT GGC CTG TAA AA, reverse: CGT GGT CAG TCA ACA TGG AT; *Nlrp3*, forward: AGC TAT GAG GTG GTG ATG TTA GTG G, reverse: CAC AAT ATT TGT ACC AGA CAG ACT TGA G; *Asc*, forward: ATG GGG CGG GCA CGA GAT G, reverse: GCT CTG CTC CAG GTC CAT CAC; *Pro-caspase-1*: forward: TGG TCT TGT GAC TTG GAG GA, reverse: TGG CTT CTT ATT GGC ACG AT; *Pro-Il-1β*, forward: GAA ATG CCA CCT TTT GAC AGT G, reverse: TGG ATG CTC TCA TCA GGA CAG. Raw data were normalized to Gapdh expression and were analyzed by the 2^-ΔΔCt^ method by calculating the relative expression to the saline control.

### Inflammasome Activation/IL-1β Release Assay

In some cultures, BMDMs were seeded at the density of 1 × x 10^6^ cells in 96-well plate. After 24 h, the cells were stimulated with 500 ng/mL of lipopolysaccharide (LPS) from *Escherichia coli* (Santa Cruz Biotechnology) and 3 h later treated with 450 μg/ml of MSU to stimulate NLRP3 inflammasome activation as described previously (Martinon et al., 2006). To evaluate effect of *trans*-Chalcone on NF-κB activation *in vitro*, BMDMs were pre-treated with *TC* at 0.1–3 μM 30 min before LPS stimulation (priming). Supernatants were collected 5 h after MSU stimulation and IL-1β concentration was quantitated by ELISA. In another experimental set, BMDMs were treated with *TC* at 0.1–3 μM 30 min after LPS priming and before MSU stimulation, in order to address the direct effect on inflammasome activation. Supernatants were also collected 5 h after MSU stimulation to assess IL-1β concentration. The data are representative of the means of three independent experiments.

Lactate dehydrogenase (LDH) release in the supernatant was used to determine cytotoxicity using LDH Cytotoxicity Assay Kit (Cayman Chemical, MI, United States) according to the manufacturer’s directions. Trypan blue exclusion test of cell viability was also performed.

### Cytokine Measurement

Mice were anesthetized and killed, and knee joint was collected and frozen with liquid nitrogen. The samples were then homogenized in 500 μl of buffer containing protease inhibitors, centrifuged (3600 rpm × 4°C × 15 min) and the supernatants were used to determine the levels of TNF-α (#88-7324-76), IL-1β (#88-8014-22), IL-6 (#88-7064-76), TGF-β (#88-8350-76), and IL-10 (#88-7105-76) by an enzyme-linked immunosorbent assay (ELISA) using eBioscience/Thermo Fisher Scientific kits. The levels of IL-1β were also assessed in BMDM culture. The results are expressed as picograms (pg) of cytokine/100 mg of tissue or pg/ml.

### NF-κB Activation

Mice were anesthetized and killed, and knee joint was collected and frozen with liquid nitrogen. The samples were then homogenized with a tissue-tearor in 500 μl of ice-cold lysis buffer (Cell Signaling). The homogenates were centrifuged (14000 rpm × 10 min × 4°C), with the supernatants used to assess the levels of total (#7836) and phosphorylated (#7834) NF-κB p65 subunit by ELISA using PathScan kits^®^ (Cell Signaling, Danvers, MA, United States) according to the manufacturer’s directions. Data were expressed as the total NF-κB p65/phospho-NF-κB ratio p65 measured at 450 nm (Multiskan GO, Thermo Fisher Scientific).

### Enzymatic Markers of Liver Injury

Blood samples were collected by cardiac puncture 15.5 h after treatment with *trans*-Chalcone at 30 mg/kg or 10 h after stimulus with acetaminophen at 650 mg/kg and added into microtubes containing anticoagulant (EDTA, 5,000 IU/mL, Sigma Chemical Co., St. Louis, MO, United States). Acetaminophen was used as positive control of liver injury as described previously (Fattori et al., 2017a). The plasma was separated by centrifugation (200 ×*g*, 10 min, 4°C). Plasma samples were processed according to the manufacturer’s instructions (Labtest Diagnóstica S.A., Lagoa Santa, Brazil) to evaluate ALT and AST levels as indicators of hepatotoxicity. Results are presented as U/L of plasma ALT or AST.

### Renal Function Tests

Blood samples were collected by cardiac puncture 15.5 h after treatment with *trans*-Chalcone at 30 mg/kg or 24 h after stimulus with diclofenac at 200 mg/kg and added into microtubes containing anticoagulant (EDTA, 5,000 IU/mL, Sigma Chemical Co., St. Louis, MO, United States). Diclofenac was used as positive control of kidney injury as described previously (Fattori et al., 2017a). The plasma was separated by centrifugation (200 ×*g*, 10 min, 4°C). Plasma samples were processed according to the manufacturer’s instructions (Labtest Diagnóstica S.A., Lagoa Santa, Brazil) to evaluate urea and creatinine levels as indicators of nephrotoxicity. Results are presented as mg/dL of plasma urea or creatinine.

### Data Analysis

Data were analyzed using GraphPad Prism statistical software (GraphPad Software, Inc., United States-500.288, version 5.0). Results are presented as means ± SEM of measurements made on 12 mice per group that were obtained in two separate experiments with 6 mice per group. For *in vitro* experiments, 6 wells were performed per group per experiment, and every experiment was repeated three times, and results are the mean of the three experiments. Two-way ANOVA was used to compare the groups and doses at all times when the parameters were measured at different times after the stimulus injection. The analyzed factors were treatments, time, and time versus treatment interaction. One-way ANOVA followed by Tukey’s test was performed for each time-point. *P* < 0.05 was considered significant.

## Results

### *Trans*-Chalcone Inhibits MSU-Induced Mechanical Hyperalgesia and Edema in a Dose-Dependent Manner

**FIGURE 1:**
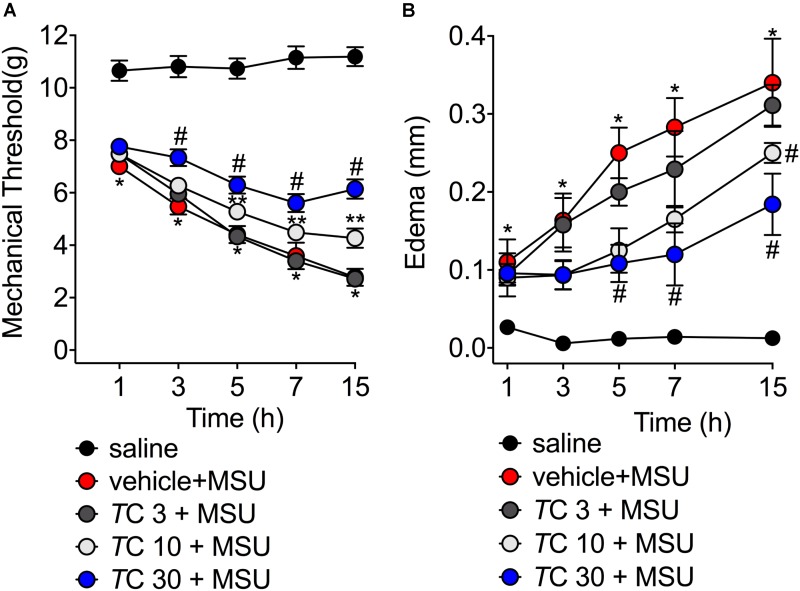
*Trans*-Chalcone inhibits MSU-induced mechanical hyperalgesia and edema in a dose-dependent manner. Mice were treated *Trans*-Chalcone (*T*C, 3, 10, or 30 mg/kg, p.o., 100 μl) or vehicle (Tween 80 20% plus saline) 30 min before MSU (100 μg/10 μl/knee) stimulus in the femur-tibial joint of swiss mice. **(A)** Mechanical hyperalgesia and **(B)** edema were evaluated 1, 3, 5, 7, and 15 h after MSU injection. Results are expressed as mean ± SEM, data represent a total of 12 mice per group that were obtained in two independent experiment with 6 mice per experiment. (^∗^*p* < 0.05 vs. control group; ^#^*p* < 0.05 vs. vehicle group, and TC 3 group, two-way ANOVA followed by Tukey’s post-test). ^∗∗^*p* < 0.05 vs. vehicle group.

Severe pain and swelling around the joint are the most clinical findings after acute gout in humans causing disability to the patients (Amaral et al., 2012; Ruiz-Miyazawa et al., 2017). Considering these features, in the first set of experiments we addressed whether *trans*-Chalcone would inhibit MSU-induced knee joint mechanical hyperalgesia and edema within 15 h after MSU injection. MSU injection induced mechanical hyperalgesia, which was not reduced by *trans*-Chalcone at the dose of 3 mg/kg in any evaluated time-points (**Figure 1A**). *Trans*-Chalcone at the dose of 10 mg/kg reduced MSU-induced mechanical hyperalgesia between 5–15 h. The dose of 30 mg/kg of *trans*-Chalcone inhibited MSU-induced mechanical hyperalgesia between 3–15 h, with significant statistical difference compared to the doses of *trans*-Chalcone of 10 mg/kg in the 15th hour post-MSU (**Figure 1A**). Only *trans*-Chalcone at 30 mg/kg inhibited MSU-induced knee joint edema in more than one-time point (5–15 h post-MSU) (**Figure 1B**). Furthermore, in a post-treatment protocol with *trans*-Chalcone (**Supplementary Figure S1**), the dose of 30 mg/kg reduced the MSU-induced knee joint mechanical hyperalgesia at 1, 3, and 15 h post-stimulus (**Supplementary Figure S1A**). The difference in edema were observed at 7 and 15 post-MSU stimulus (**Supplementary Figure S1B**).

### *Trans*-Chalcone Inhibits MSU-Induced Leukocyte Recruitment to the Knee Joint and Synovitis

**FIGURE 2:**
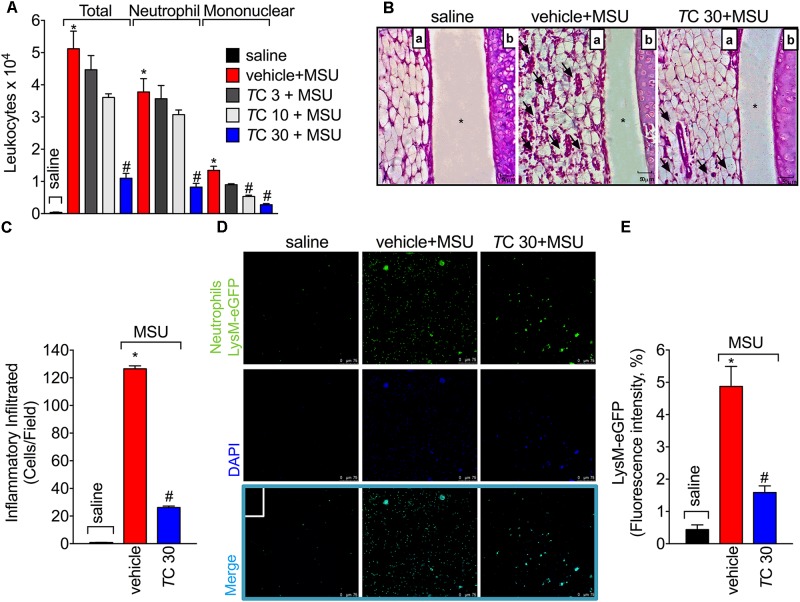
*Trans*-Chalcone inhibits MSU-induced leukocyte recruitment in a dose-dependent manner. Mice were treated *Trans*-Chalcone (*T*C, 3, 10, or 30 mg/kg, p.o., 100 μl) or vehicle (Tween 80 20% plus saline) 30 min before MSU (100 μg/10 μl/knee) stimulus in the femur-tibial joint of swiss mice **(A)** Fifteen hours after MSU, knee joint wash was collected for counting total leukocytes, neutrophils, and mononuclear cells. **(B,C)** Fifteen hours after MSU, knee joint was collected for histopathological analysis by HE staining. Original magnification image of 400× of groups: saline, vehicle+MSU and *T*C30+MSU. For panel **(B)** arrows indicate leukocyte infiltration, (a) synovial tissue, (b) cartilage tissue, and ^∗^ joint space. Panel **(C)** shows the total score of inflammatory infiltrate (synovitis). **(D,E)** Fifteen hours after MSU, knee joint washes of MSU-stimulated LysM-GFP^+^ mice were collected for the determination of LysM-GFP^+^ neutrophil recruitment by confocal microscopy. Original magnification 200×. Panel **(E)** shows the percentagem of LysM-eGFP+ fluorescence. Results are expressed as mean ± SEM, data represent a total of 12 mice per group that were obtained in two independent experiment with 6 mice per experiment. (^∗^*p* < 0.05 vs. control group; ^#^*p* < 0.05 vs. vehicle, one-way ANOVA followed by Tukey’s post-test).

Leukocyte recruitment the knee joint, especially of neutrophils, is a hallmark of gout arthritis pathology (Amaral et al., 2012; Fattori et al., 2016; Ruiz-Miyazawa et al., 2017). Thus, the effect of *trans*-Chalcone on MSU-induced leukocyte recruitment was evaluated. Only *trans*-Chalcone at the dose of 30 mg/kg reduced MSU-induced total leukocyte (**Figure 2A**), neutrophil (**Figure 2A**), and mononuclear cell recruitment (**Figure 2A**). However, the dose of 10 mg/kg of *trans*-Chalcone inhibited MSU-induced mononuclear cell recruitment (**Figure 2A**). Considering the results of **Figures 1**, **2A**, the dose of 30 mg/kg of *trans*-Chalcone was selected for the next experiments. In agreement with **Figures 1**, **2A**, the histopathological finding shows that *trans*-Chalcone reduced inflammatory cell recruitment to the knee joint (**Figures 2B,C**), suggesting a reduction in the synovitis, an inflammation of synovial membrane. Furthermore, by using LysM-eGFP mice as another approach to investigate MSU-induced neutrophil recruitment, we show that *trans*-Chalcone reduced the infiltration of LysM-eGFP^+^ cells that includes neutrophils and monocytes/macrophages (**Figures 2D,E**), as observed by reduced intensity of fluorescence (**Figures 2D,E**). Importantly, *trans*-Chalcone at 30 mg/kg did not induce liver injury (**Supplementary Figures S2A,B**) or kidney injury (**Supplementary Figures S2C,D**). These side effects are commonly associated with the use of analgesics such as acetaminophen (APAP, paracetamol) (Hohmann et al., 2015) or anti-inflammatory drugs such as diclofenac (Fattori et al., 2017a).

### *Trans*-Chalcone Inhibits MSU-Induced Oxidative Stress

**FIGURE 3:**
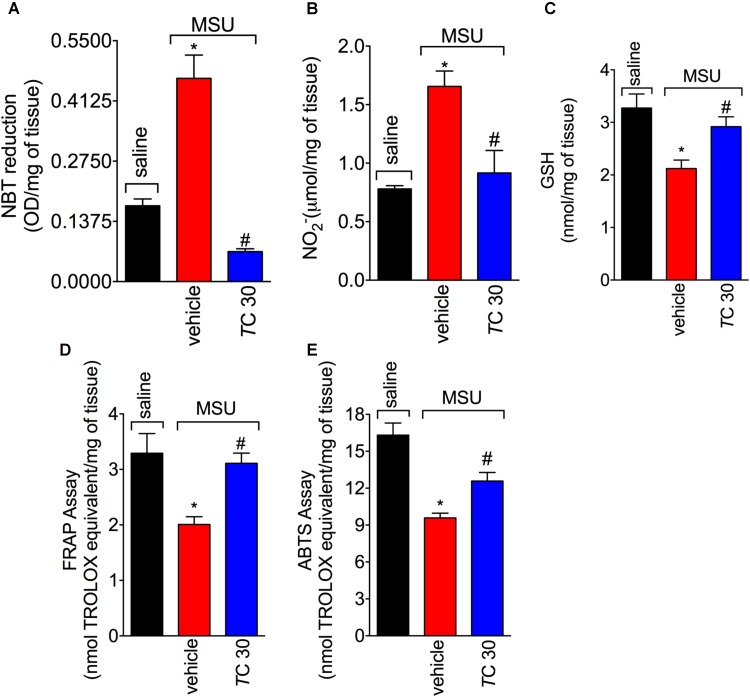
*Trans*-Chalcone inhibits MSU-induced oxidative stress. Mice were treated *Trans*-Chalcone (*T*C, 30 mg/kg, p.o., 100 μl) or vehicle (Tween 80 20% plus saline) 30 min before MSU (100 μg/10 μl/knee) stimulus in the femur-tibial joint of swiss mice. Fifteen hours after MSU, knee joint samples were collected and the oxidative stress was assessed by measuring: **(A)** superoxide anion (NBT assay), **(B)** NO_2_^-^ production Griess assay), **(C)** GSH levels, **(D)** Ferring reducing power (FRAP assay), and **(E)** ABTS scavenging ability (ABTS assay). Results are expressed as mean ± SEM, data represent a total of 12 mice per group that were obtained in two independent experiment with 6 mice per experiment. (^∗^*p* < 0.05 vs. control group; #*p* < 0.05 vs. vehicle, one-way ANOVA followed by Tukey’s post-test).

**FIGURE 4:**
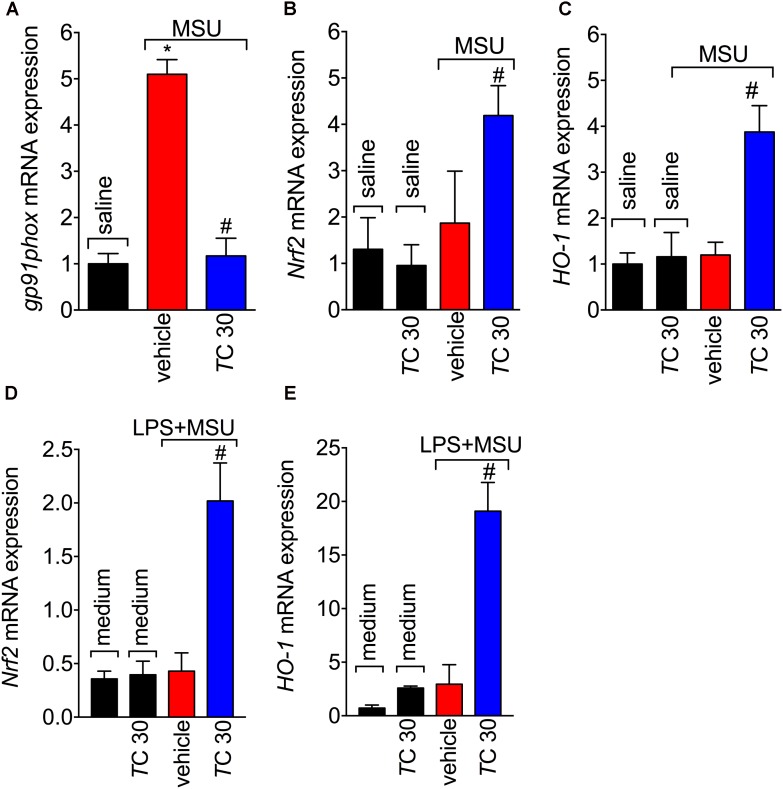
*Trans*-Chalcone inhibits MSU-induced *gp91phox* mRNA expression and increases *Nrf2* and *Ho-1* mRNA expression. Mice were treated *Trans*-Chalcone (*T*C, 30 mg/kg, p.o., 100 μl) or vehicle (Tween 80 20% plus saline) 30 min before MSU (100 μg/10 μl/knee) or sterile saline (10 μl/knee) stimulus in the femur-tibial joint of swiss mice. Fifteen hours after MSU, knee joint samples were collected for the determination of the mRNA expression of: **(A)** *gp91phox*, **(B)** *Nrf2*, and **(C)** *HO-1* by RT-qPCR. 3 × 10^6^ BMDMs were seeded in 6 well plate and pre-treated with 3 μM of *trans*-chalcone 30 min before 500 ng/mL of LPS for priming over 3 h, followed by MSU (450 μg/mL) activation. After 5 h, the cells were collected for determination of mRNA expression of: **(D)** *Nrf2* and **(E)** *HO-1* by RT-qPCR. Results are expressed as mean ± SEM, data represent a total of 12 mice per group that were obtained in two independent experiment with 6 mice per experiment or the mean of 6 wells per group per experiment with three independent experiments for *in vitro* assays. (^∗^*p* < 0.05 vs. control group; #*p* < 0.05 vs. vehicle, one-way ANOVA followed by Tukey’s post-test).

Given the role of reactive oxygen and nitrogen species in pain processing and inflammation (Gloire and Piette, 2009; Martinon, 2010; Fattori et al., 2016; Grace et al., 2016) and that flavonoids are recognized by their antioxidant activity (Verri et al., 2012), the effect of *trans*-Chalcone in MSU-induced oxidative stress was investigated. *Trans*-Chalcone inhibited MSU-induced production of superoxide anion (NBT assay; **Figure 3A**) and nitric oxide (NO_2_^-^ assay; **Figure 3B**) in knee joint samples. In line with these results, *trans*-Chalcone also restored the endogenous antioxidant capacity of samples by preventing depletion in the GSH levels (**Figure 3C**) and increasing the FRAP (FRAP assay, **Figure 3D**) and ABTS free radical scavenging ability (ABTS assay, **Figure 3E**) that were reduced by MSU injection. ABTS in an effective assay to screen the presence of antioxidant compounds and FRAP assay identifies the reducing ability exerted by antioxidants (Menghini et al., 2018). Thus, both assays were essential tools to demonstrate the *in vivo* antioxidant effect of *trans*-chalcone in gouty arthritis. Corroborating the results of oxidative stress status, *trans*-Chalcone reduced MSU-induced *gp91phox* mRNA expression (**Figure 4A**), a NADPH oxidase subunit. *Trans*-Chalcone also increased the mRNA expression of the antioxidant transcription factor *Nrf2* (**Figure 4B**) and its downstream target HO-1 (**Figure 4C**). Importantly, the treatment with *trans*-chalcone in saline group (without MSU stimulus) did not affect the mRNA expression of both genes, pointing that *trans*-chalcone does not have an effect *per se* over Nrf2 and HO-1 mRNA expression (**Figures 4B,C**). Furthermore, in an *in vitro* system approach using BMDM culture, we also observed that the pre-treatment (30 min) with *trans*-chalcone in a concentration of 3 μM enhanced *Nrf2* as well as *Ho-1* mRNA expression under LPS plus MSU stimuli. The treatment of naive BMDM with *trans*-chalcone did not alter *Nrf2* (**Figure 4D**) and *Ho-1* (**Figure 4E**) mRNA expression, which corroborates the *in vivo* data. Therefore, *trans*-Chalcone inhibited MSU-induced pro-oxidant enzymes and increases antioxidant sensitive pathways in the knee joints, confirming an important effect of this flavonoids in the redox biology of gouty arthritis.

### *Trans*-Chalcone Modulates *in vivo* and *in vitro* MSU-Induced Cytokine Production

**FIGURE 5:**
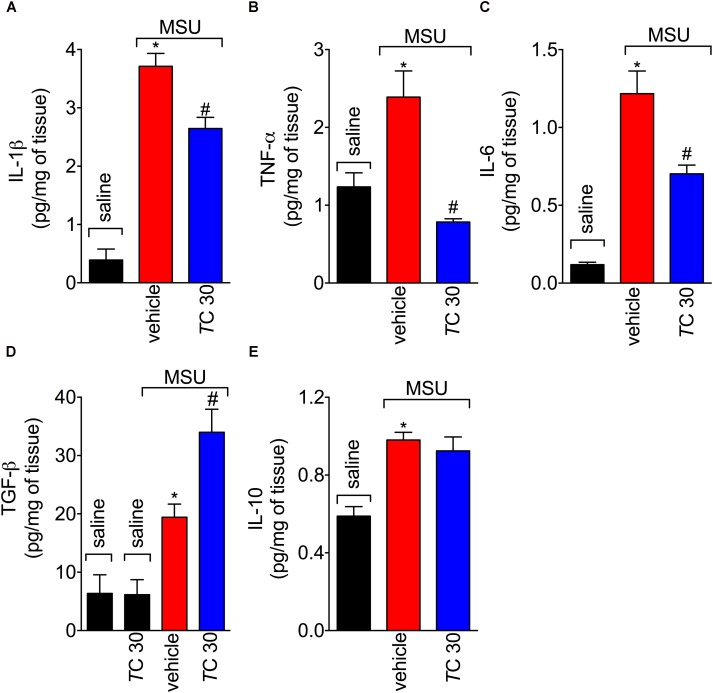
*Trans*-Chalcone modulates MSU-induced cytokine production *in vivo*. Mice were treated *trans*-Chalcone (*T*C, 30 mg/kg, p.o., 100 μl) or vehicle (Tween 80 20% plus saline) 30 min before MSU (100 μg/10 μl/knee) stimulus in the femur-tibial joint of swiss mice. Fifteen hours after MSU, knee joint samples were collected for the determination of **(A)** IL-1β, **(B)** TNF-α, **(C)** IL-6, **(D)** TGF-β, and **(E)** IL-10 production by ELISA. Results are expressed as mean ± SEM, data represent a total of 12 mice per group that were obtained in two independent experiment with 6 mice per experiment. (^∗^*p* < 0.05 vs. control group; #*p* < 0.05 vs. vehicle, one-way ANOVA followed by Tukey’s post-test).

**FIGURE 6:**
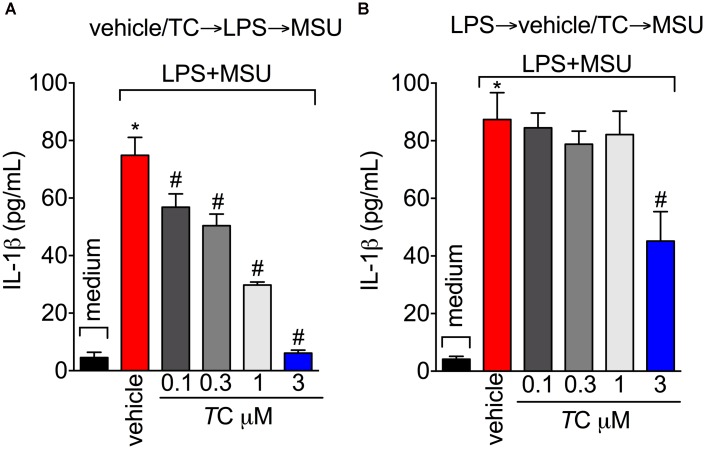
*Trans*-Chalcone reduces MSU-induced IL-1β maturation *in vitro*. **(A)** To assess the effect of *trans*-Chalcone on NF-κB activation, BMDMs were pre-treated with 0.1–3 μM before 500 ng/mL of LPS [i.e., before priming, panel **(A)** and after 3 h were secondarily stimulated with MSU (450 μg/ml, i.e., activation]. **(B)** To assess the direct effect of *trans*-Chalcone on inflammasome activation, LPS-primed BMDMs were treated with 0.1–3 μM 30 min before MSU stimulation (i.e., after priming and before activation). Results are expressed as mean ± SEM, data represent the mean of 6 wells per group per experiment with three independent experiments. (^∗^*p* < 0.05 vs. control group; #*p* < 0.05 vs. vehicle, one-way ANOVA followed by Tukey’s post-test).

Next, the effect of *trans*-Chalcone on MSU-induced pro-inflammatory cytokine production was investigated considering the importance of cytokines to gout arthritis pathology (Dalbeth et al., 2016; Fattori et al., 2016). Treatment with *trans*-Chalcone reduced *in vivo* MSU-induced IL-1β (**Figure 5A**), TNFα (**Figure 5B**), and IL-6 (**Figure 5C**) production. Moreover, *trans*-Chalcone also increased the levels of the anti-inflammatory cytokine TGF-β (**Figure 5D**) without changing the levels of IL-10 in knee joints (**Figure 5E**). Regarding TGF-β levels, *trans*-Chalcone did not alter the levels of this anti-inflammatory cytokine *per se* (**Figure 5D**). As IL-1β production in its mature form depends on priming and activation (Martinon et al., 2006) and some flavonoids can potentially inhibit NF-κB activation (Vicentini et al., 2001) and IL-1β maturation (Domiciano et al., 2017), we investigated whether or not *trans*-Chalcone reduced IL-1β production by targeting LPS-induced NF-κB activation (LPS/priming) and/or interfering with inflammasome activation (MSU/activation). BMDM pre-treatment (before priming with LPS) with *trans*-Chalcone at the concentrations of 0.3, 1, and 3 μM reduced MSU-induced release of IL-1β in the supernatant of cell culture (**Figure 6A**). In a post-treatment protocol, after priming the BMDM with LPS and before secondary stimulation with MSU, the treatment with *trans*-Chalcone at 3 μM also reduced MSU-induced IL-1β release by LPS-primed BMDM (**Figure 6B**). Thus, these data indicate that *trans*-Chalcone can either inhibit NF-κB activation (before priming with LPS; **Figure 6A**) and directly interfere with inflammasome activation in a concentration-dependent manner (activation, treatment after LPS, but before MSU; **Figure 6B**). However, the effect of *trans*-Chalcone was more prominent in inhibiting the priming with LPS (lower concentrations of *trans*-Chalcone) than the activation by MSU (only the highest concentration of *trans*-Chalcone), which indicates *trans*-Chalcone presents a more prominent effect in inhibiting NF-κB activation than inflammasome activation as a main mechanism. Importantly, none of the concentrations used herein (up to 3 μM) of *trans*-Chalcone reduced cell viability as per LDH analysis (**Supplementary Figure S3A**) and Trypan Blue (**Supplementary Figure S3B**).

### *Trans*-Chalcone Inhibits MSU-Induced *Nlrp3*, *Asc*, *Pro-caspase-1*, and *Pro-Il-1β* mRNA Expression, and NF-κB Activation

**FIGURE 7:**
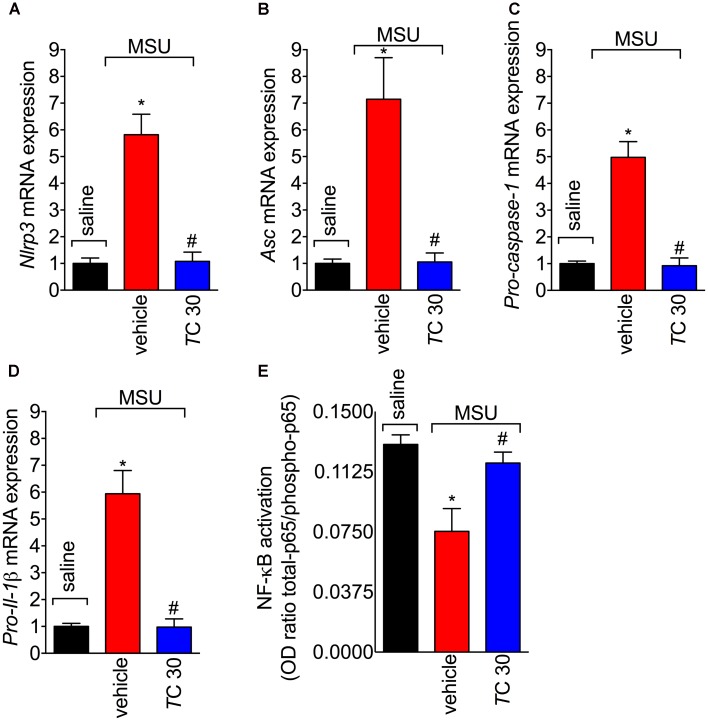
*Trans*-Chalcone decreases MSU-induced *Nlrp3*, *Asc*, *Pro-caspase-1*, and *Pro-Il-1β* mRNA expression, and NF-κB activation. Mice were treated with *trans*-Chalcone (*T*C, 30 mg/kg, p.o., 100 μl) or vehicle (Tween 80 20% plus saline) 30 min before MSU (100 μg/10 μl/knee) stimulus in the femur-tibial joint of swiss mice. Fifteen hours after MSU, knee joint was collected for determination of mRNA expression of **(A)** *Nlrp3*, **(B)**, *Asc*, **(C)** *Pro-caspase-1*, **(D)** *Pro-Il-* by RT-qPCR, and **(E)** NF-κB activation by ELISA. Results are expressed as mean ± SEM, data represent a total of 12 mice per group that were obtained in two independent experiment with 6 mice per experiment. (^∗^*p* < 0.05 vs. control group; #*p* < 0.05 vs. vehicle, one-way ANOVA followed by Tukey’s post-test).

The assembly of NLRP3 inflammasome is a crucial mechanism of gout arthritis that is dependent on NF-κB activation, which is a transcription factor essential to inducing the expression of inflammasome components and pro-IL-1β (Martinon et al., 2006; Amaral et al., 2012; Dalbeth et al., 2016). Given *trans*-Chalcone reduced *in vivo* and *in vitro* IL-1β production, the effect of *trans*-Chalcone on the mRNA expression of inflammasome components *Nlrp3*, *Asc*, and *Pro-caspase-1*, and *Pro-Il-1β* was investigated. Treatment with *trans*-Chalcone reduced the mRNA expression of all inflammasome components *Nlrp3* (**Figure 7A**), *Asc* (**Figure 7B**), *Pro-caspase-1* (**Figure 7C**), and *Pro-Il-1β* (**Figure 7D**). Further, MSU induced the activation of NF-κB as observed by a decrease of total-p65/phosphorylated-p65 OD ratio (**Figure 7E**). The decrease in the ratio demonstrates the increase in the p65 subunit phosphorylation, therefore, indicating activation of NF-κB. In turn, *trans*-Chalcone treatment inhibited MSU-induced NF-κB activation (**Figure 7E**). Thus, these data confirmed that the effect of *trans*-Chalcone in gout arthritis is dependent on inhibiting NF-κB activation, which will then result in reducing inflammasome platform expression.

## Discussion

In this study, we show that the precursor of flavonoids in plants, *trans*-Chalcone, ameliorates experimental gout arthritis in mice. This effect is related to the reduction of MSU-induced oxidative stress, NF-κB activation and thereby inhibition of inflammasome expression and production of IL-1β in its mature form. *Trans*-Chalcone also increased the production of the anti-inflammatory cytokine TGF-β. It is also important to highlight that, to our knowledge, this is the first demonstration that *trans*-Chalcone has analgesic properties and also inhibits MSU-induced joint inflammation.

The management of gout acute flares lies on the use of corticosteroids, AINEs, colchicine, and biological agents (Rees et al., 2014). These single target therapies possess limit analgesic efficacy and when effective often produce several undesirable side effects in gout arthritis, a disease with multiple physiopathological mechanisms (Rees et al., 2014). Flavonoids are widely known for their high spectrum of biological action and low toxicity, which are explained by the fact that flavonoids are multi-target drugs, inhibiting but not abolishing endogenous pathways (Verri et al., 2012). Herein, we show that *trans*-Chalcone did not induce toxicity *in vivo* and *in vitro*, indicating a safe preclinical profile. Although the literature did not define the *trans*-Chalcone pharmacokinetic profile after oral administration, we could observe that it inhibited gout-induced pain and inflammation as a pre-treatment and post-treatment, which reinforces its therapeutic potential. The antioxidant activity is a hallmark of the activity of flavonoids (Verri et al., 2012) and in fact, the reduction of oxidative and nitrosative stress are important mechanisms by which flavonoids (Anjaneyulu and Chopra, 2003; Al-Rejaie et al., 2015; Ahmed et al., 2016; Pinho-Ribeiro et al., 2016) and other molecules (Fattori et al., 2015, 2017a; Singh and Vinayak, 2015) act. Moreover, injection of a superoxide anion donor, peroxynitrite, or intrathecal delivery of ROS elicits pain behavior in mice (Wang et al., 2004; Fattori et al., 2015, 2017b) indicating that oxidative stress plays important role in pain processing (Grace et al., 2016). Neutrophils produce ROS upon recognition of MSU crystals (Desaulniers et al., 2001) and, in turn, ROS contribute to neutrophil recruitment (Hattori et al., 2010; Sakai et al., 2012). Moreover, nitric oxide quickly react with ROS to produce peroxynitrite and there is a correlation between increased NO generation and gouty arthritis progression (Chen et al., 2004).

The antioxidant capacity of phenolic compounds is related to the ability to generate a stable molecule after donating electrons. An important structural determinant of the antioxidant capacity of flavonoids is attributed to hydroxyl C4 and C3 (Verri et al., 2012). The structure-activity relationship of *trans*-Chalcone indicates that it does not possess antioxidant properties (Martinez et al., 2017a). In fact, using a cell free *in vitro* system, *trans*-Chalcone did not show antioxidant activity in the ABTS (electron transfer), DPPH (hydrogen transfer), iron chelation and superoxide anion (free radical) assays (Martinez et al., 2017a). Thus, electronic transfer of electrons, chelating transition metals or direct scavenging activity are not mechanisms by which *trans*-Chalcone reduces oxidative stress. However, *in vivo* data show that *trans*-Chalcone displays antioxidant effect observed by an increase of antioxidant defense and reduction of ROS (Karkhaneh et al., 2016; Singh et al., 2016; Martinez et al., 2017a,b). We also observed that *trans*-Chalcone maintained the tissue antioxidant activities such as FRAP reducing and ABTS radical scavenging ability in knee joint samples, and promoted glutathione system improvement, besides inhibiting the production of ROS and reducing nitrite levels. Therefore, the antioxidant effect of *trans*-Chalcone is not related to a direct chemical antioxidant structure, but rather is dependent on its anti-inflammatory effects and the activation of Nrf2 signaling pathway (Wu et al., 2014; Martinez et al., 2017b). In fact, *trans*-Chalcone also inhibited gp91^phox^ mRNA expression and induced the mRNA expression of the antioxidant transcription factor *Nrf2* and its downstream target *HO-1*. Our results corroborate other reports showing that trans-Chalcone acts on the antioxidant transcription factor Nrf2 (Wu et al., 2014; Martinez et al., 2017b) and reducing Kelch-like ECH-associated protein 1 (Keap1) activity (Kobayashi et al., 2006). However, it is unlikely that in the present experimental condition *trans*-Chalcone is acting by increasing Nrf2 and HO-1 *per se*, but rather, this increase seems to occur only during inflammation.

In addition to antioxidant effects, Nrf2/HO-1 signaling is an important analgesic pathway (Steiner et al., 2001; Carcole et al., 2014). In fact, co-treatment with an HO-1 inducer increases the analgesic effects of opioids and cannabinoids through the activation of cGMP/PKG/ATP-sensitive potassium channel pathway in the CFA-induced inflammatory pain model (Carcole et al., 2014). Thus, the increase of *Nrf2*/*Ho-1* mRNA expression contributed to the reduction of MSU-induced oxidative stress and pain in this study.

The main innate immune event in MSU crystals-induced inflammation is the NLRP3-dependent maturation of IL-1β (Martinon et al., 2006; Amaral et al., 2012). This mechanism accounts for neutrophil recruitment and pain (Amaral et al., 2012). In response to MSU crystals, other macrophages-derived mediators such as TNF-α also drive neutrophil migration toward the tissue (Amaral et al., 2016). Moreover, targeting TNF-α, IL-1β, or IL-33 in rheumatic diseases reduce neutrophil recruitment and pain (Verri et al., 2008, 2010; Amaral et al., 2012, 2016). *Trans*-Chalcone reduced *in vivo* MSU-induced TNF-α, IL-1β, and IL-6; and *in vitro* maturation of IL-1β. Therefore, the reduction of these cytokines certainly contributed to the inhibition of neutrophils recruitment. This is an important finding because neutrophil recruitment is a hallmark of the acute phases of all rheumatic diseases (Fattori et al., 2016) and during gout flares, they are the predominant cell population in the synovial fluid (Mitroulis et al., 2013). After recognition of MSU crystals by neutrophils, they degranulate (Popa-Nita et al., 2007) and undergo NETosis (Mitroulis et al., 2011). Moreover, activated neutrophils produce IL-1β, TNF-α, and PGE_2_ that contribute to pain (Cunha et al., 2008; Verri et al., 2010; Amaral et al., 2012, 2016). IL-1β and TNF-α also activate nociceptor neuron and thereby producing pain (Jin and Gereau, 2006; Binshtok et al., 2008). Regarding IL-1β, two steps are required for the production and release of its mature form (Martinon et al., 2006). The first step is related to downstream signaling pathways that depend on NF-κB activation or a priming step, whereas the second step is related to inflammasome assembly *per se* that depends on the phagocytosis of MSU crystals and lysis of phagolysosome releasing cathepsin that activates NLRP3 (Martinon et al., 2006; Amaral et al., 2012; Latz et al., 2013). Thus, naturally occuring molecules (such as flavonoids) that target priming and/or activation signal to inflammasome assembly without the side effect of the current therapies are likely to be highly attractive as an analgesic approach in gout arthritis. In fact, the flavonoid quercetin that inhibits NLRP3 inflammasome and ASC speck formation in mouse vasculitis (Domiciano et al., 2017) and reduces pain in experimental gout arthritis (Ruiz-Miyazawa et al., 2017). In the present study, pre-treated BMDMs (treatment before LPS stimulus, i.e., priming) with *trans*-Chalcone reduced MSU-induced IL-1β production suggesting that this flavonoid reduced NF-κB activation (priming). In fact, this effect was further confirmed by *in vivo* data as demonstrated by an increase of NF-κB OD ratio by ELISA. In agreement with the present findings that *trans*-chacone inhibition of NF-κB activation in gout arthritis, evidence demonstrated that *trans*-Chalcone reduces NF-κB activation in murine retina (Lamoke et al., 2011). Thus, the inhibition of NF-κB activation explains the reduced levels of the pro-inflammatory cytokines observed. Moreover, *trans*-Chalcone reduced MSU-induced IL-1β production in the supernatant of LPS-primed BMDMs (that already received priming) indicating that it may also interfere with inflammasome assembly. In fact, other naturally occuring molecules, such as the flavonoids quercetin (Domiciano et al., 2017), apigenin (Zhang et al., 2014), and oroxylin A (Zhou et al., 2017); and the sesquiterpene lactone parthenolide (Juliana et al., 2010) have the ability to interfere with inflammasome assembly. This mecanim is related to the inhibition of ASC speck formation (Zhang et al., 2014; Domiciano et al., 2017; Zhou et al., 2017) or direct targeting of active site of caspase-1 (Juliana et al., 2010). In addition to inhibiting pro-inflammatory cytokines production, *trans*-Chalcone also increased the level of the anti-inflammatory cytokine TGF-β. This anti-inflammatory cytokine plays an important role in the resolution phase of MSU-induced inflammation (Scanu et al., 2012) by promoting efferocytosis of apoptotic neutrophils (Vieira et al., 2017). Of note, the fact that *trans*-Chalcone sustained the production of IL-10 and inhibited pro-inflammatory cytokine production is a relevant finding given the anti-hyperalgesic and anti-inflammatory properties of IL-10 and that, in general, anti-inflammatory cytokines are co-released during inflammation to limit the inflammatory response (Verri et al., 2006). Therefore, the reduction of neutrophil recruitment and pro-inflammatory cytokine production, the increase of anti-inflammatory cytokine TGF-β, and the maintenance of anti-hyperalgesic cytokine IL-10 contribute to the analgesic effect of *trans*-Chalcone.

Concluding, we demonstrated that *trans*-Chalcone ameliorates MSU-induced pain and inflammation. *Trans*-Chalcone reduced MSU-induced oxidative stress and thereby demonstrating that this molecule possesses antioxidant effect by modulating Nrf2/HO-1 signaling pathway. We also demonstrated that *trans*-Chalcone reduced MSU-induced inflammasome assembly and NF-κB activation. As a consequence, it was also observed a reduction of IL-1β, TNF-α, and IL-6 production. Interestingly, *trans*-Chalcone increased the level of the anti-inflammatory cytokine TGF-β and sustained the levels of the anti-hyperalgesic cytokine IL-10. To our knowledge, this is the first report on the analgesic effect of *trans*-Chalcone. Therefore, *trans*-Chalcone displays a safe preclinical profile with analgesic, antioxidants, and anti-inflammatory properties.

## Author Contributions

LS-F, KR-M, RC, and WV conceived and designed the experiments. LS-F, KR-M, FP-R, VF, TZ, SB-G, SB, and TC performed the experiments. LS-F, KR-M, FP-R, VF, TZ, SB-G, SB, TC, RC, and WV contributed to collection of data and analysis. JA-F, TC, FC, RC, and WV contributed to reagents, materials, and analysis tools. LS-F, KR-M, VF, and WV wrote the original draft. LS-F, KR-M, VF, RC, and WV contributed to writing-review and editing. All authors contributed to manuscript revision and read and approved the final version of the manuscript.

## Conflict of Interest Statement

The authors declare that the research was conducted in the absence of any commercial or financial relationships that could be construed as a potential conflict of interest. The reviewer GO and handling Editor declared their shared affiliation.
