# Supplementary material for: Trans-Chalcone Attenuates Pain and Inflammation in Experimental Acute Gout Arthritis in Mice
Source: Front Pharmacol. 2018 Oct 2;9:1123. doi: 10.3389/fphar.2018.01123 (PMC6176465; doi:10.3389/fphar.2018.01123)
Supplement: Supplementary file 1 [file Presentation_1.pdf]

Trans-Chalcone attenuates pain and inflammation in experimental acute gout arthritis in mice

Larissa Staurengo-Ferrari[1,f], Kenji W. Ruiz-Miyazawa[1,f], Felipe A. Pinho-Ribeiro[1], Victor Fattori[1], Tiago H. Zaninelli[1], Stephanie Badaro-Garcia[1], Sergio M. Borghi[1], Thacyana T. Carvalho[1], Jose C. Alves-Filho[2], Thiago M. Cunha[2], Fernando Q. Cunha[2], Rubia Casagrande[3], and Waldiceu A. Verri, Jr.[1,*]

[1]Departamento de Ciências Patológicas, Universidade Estadual de Londrina-UEL, Londrina, Paraná, PR, Brazil.
[2]Department of Pharmacology, Ribeirão Preto Medical School, University of São Paulo, Ribeirão Preto, São Paulo, SP, Brazil
[3]Departamento de Ciências Farmacêuticas, Universidade Estadual de Londrina-UEL, Londrina, Paraná, PR, Brazil.

[f]These authors contributed equally.

*Correspondence:
Waldiceu A. Verri Jr
waldiceujr@yahoo.com.br; waverri@uel.br

Running title: *Trans*-chalcone attenuates gout arthritis

SUPPLEMENTARY DATA

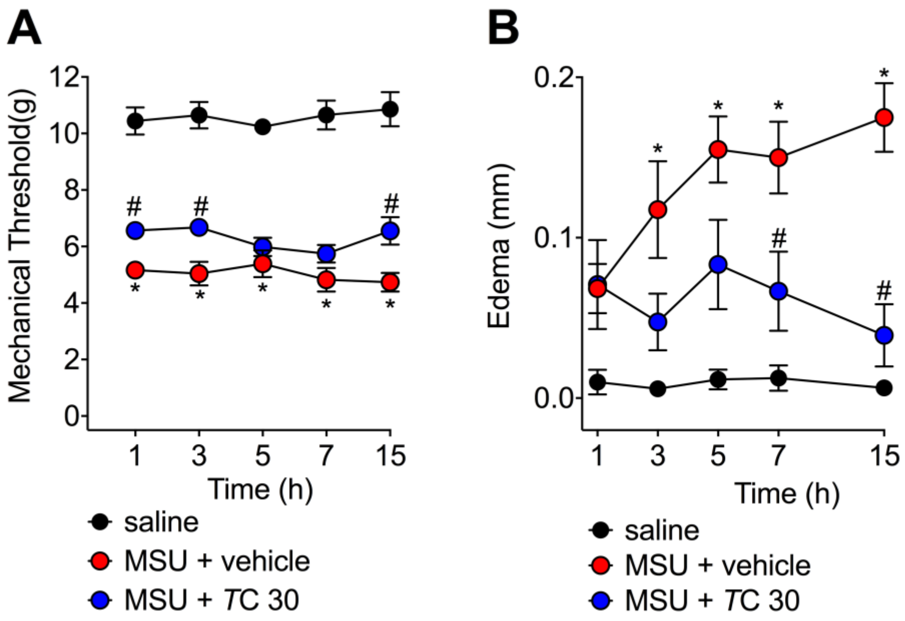

Figure S1. Effect of trans-chalcone post-treatment in MSU-induced mechanical hyperalgesia and edema. Mice were treated *Trans*-Chalcone (*T*C, 30 mg/kg, p.o., 100 µl) or vehicle (Tween 80 20% plus saline) 30 minutes after MSU (100 µg/10 µl/knee) or saline stimulus in the femur-tibial joint of swiss mice. (**A**) Mechanical hyperalgesia and (**B**) edema were evaluated 1, 3, 5, 7, and 15h after MSU injection. Results are expressed as mean ± SEM, data represent a total of 12 mice per group that were obtained in two independent experiment with 6 mice per experiment. (*$p < 0.05$ vs. control group; #$p < 0.05$ vs. vehicle group, two-way ANOVA followed by Tukey's post-test).

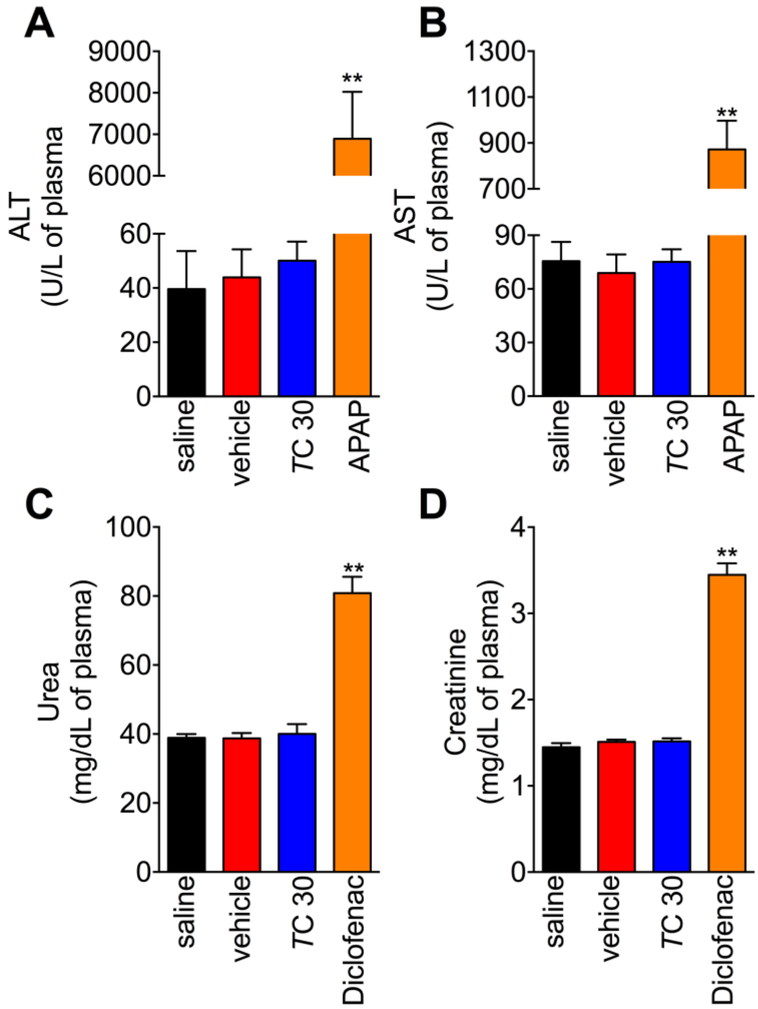

Figure S2. *Trans*-chalcone does not induce kidney or liver injury. Blood was collected 15.5h after treatment with *trans*-chalcone (*T*C, 30 mg/kg, p.o.) or vehicle (Tween 80 20% plus saline) to assess: (**A**) alanine transaminase (ALT), (**B**) aspartate aminotransferase (AST) levels in plasma samples. Acetaminophen stimulus (APAP, 650 mg/kg, p.o.) was used as a control drug for liver injury and samples were collected after 10h after stimulus. (**C**) Urea and (**D**) creatinine levels in plasma samples. Diclofenac stimulus (200 mg/kg, p.o) was used as a control drug for kidney injury and samples were collected 24h after stimulus. Results are expressed as mean ± SEM, data represent a total of 12 mice per group that were obtained in two independent experiment with 6 mice per experiment. (**$p < 0.05$ vs. all groups, one ANOVA followed by Tukey's post-test).

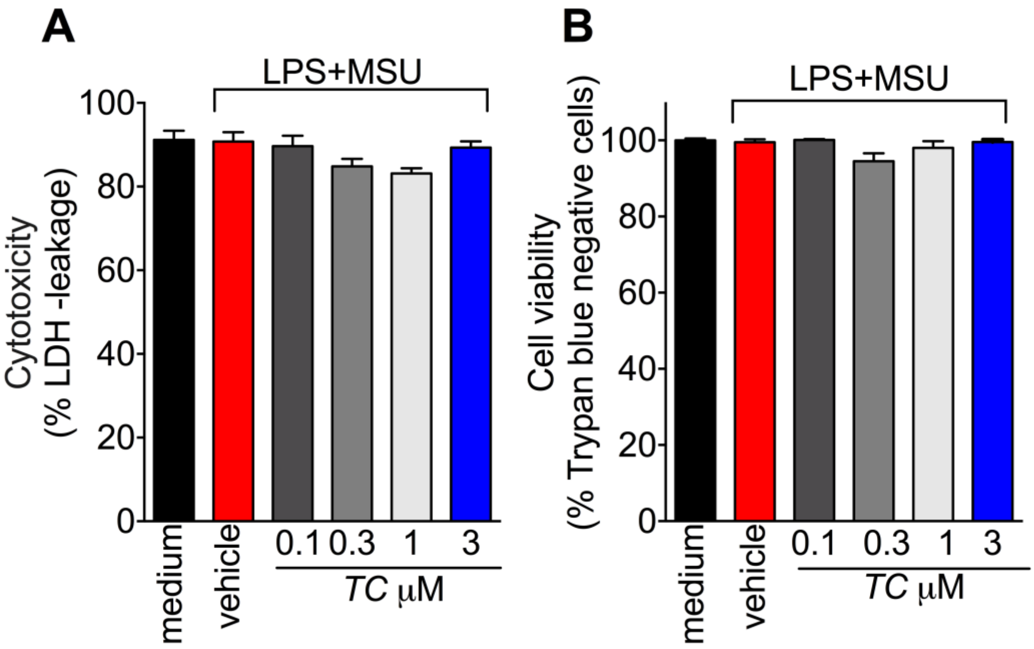

Figure S3. *Trans*-chalcone does not induce cytotoxicity or alters cell viability. BMDMs were pre-treated with 0.1-3 μM before 500 ng/mL of LPS (*before* priming) and after 3h were secondarily stimulated with MSU (450 mg/ml, activation). (**A**) Supernatants were collected 5h after MSU stimulation in BMDMs cells to assess LDH levels and (**B**) Trypan Blue assay to determine cell viability. The results were expressed as % of LDH release or dead cells from total cells counted by comparing with the positive control (vehicle group).
